# Measures of Autozygosity in Decline: Globalization, Urbanization, and Its Implications for Medical Genetics

**DOI:** 10.1371/journal.pgen.1000415

**Published:** 2009-03-13

**Authors:** Michael A. Nalls, Javier Simon-Sanchez, J. Raphael Gibbs, Coro Paisan-Ruiz, Jose Tomas Bras, Toshiko Tanaka, Mar Matarin, Sonja Scholz, Charles Weitz, Tamara B. Harris, Luigi Ferrucci, John Hardy, Andrew B. Singleton

**Affiliations:** 1 Laboratory of Neurogenetics, Intramural Research Program, National Institute on Aging, Bethesda, Maryland, United States of America; 2 Laboratory of Epidemiology, Demography and Biometry, Intramural Research Program, National Institute on Aging, Maryland, United States of America; 3 Unidad de Genética Molecular, Departamento de Genómica y Proteómica, Instituto de Biomedicina de Valencia-CSIC, Valencia, Spain; 4 Department of Molecular Neuroscience and Reta Lila Weston Laboratories, Institute of Neurology, London, United Kingdom; 5 Longitudinal Studies Section, Intramural Research Program, National Institute on Aging, Bethesda, Maryland, United States of America; 6 Department of Anthropology, Biological Anthropology Program, Temple University, Philadelphia, Pennsylvania, United States of America; 7 Department of Public Health Sciences, Center for Public Health Genomics, University of Virginia, Charlottesville, Virginia, United States of America; Queensland Institute of Medical Research, Australia

## Abstract

This research investigates the influence of demographic factors on human genetic sub-structure. In our discovery cohort, we show significant demographic trends for decreasing autozygosity associated with population variation in chronological age. Autozygosity, the genomic signature of consanguinity, is identifiable on a genome-wide level as extended tracts of homozygosity. We identified an average of 28.6 tracts of extended homozygosity greater than 1 Mb in length in a representative population of 809 unrelated North Americans of European descent ranging in chronological age from 19–99 years old. These homozygous tracts made up a population average of 42 Mb of the genome corresponding to 1.6% of the entire genome, with each homozygous tract an average of 1.5 Mb in length. Runs of homozygosity are steadily decreasing in size and frequency as time progresses (linear regression, *p*<0.05). We also calculated inbreeding coefficients and showed a significant trend for population-wide increasing heterozygosity outside of linkage disequilibrium. We successfully replicated these associations in a demographically similar cohort comprised of a subgroup of 477 Baltimore Longitudinal Study of Aging participants. We also constructed statistical models showing predicted declining rates of autozygosity spanning the 20th century. These predictive models suggest a 14.0% decrease in the frequency of these runs of homozygosity and a 24.3% decrease in the percent of the genome in runs of homozygosity, as well as a 30.5% decrease in excess homozygosity based on the linkage pruned inbreeding coefficients. The trend for decreasing autozygosity due to panmixia and larger effective population sizes will likely affect the frequency of rare recessive genetic diseases in the future. Autozygosity has declined, and it seems it will continue doing so.

## Author Summary

Population geneticists use genetic markers to quantify and compare levels of inbreeding in populations and identify disease-associated loci; epidemiologists utilize demographic factors to quantify disease risk modifiers. Our research group sought to investigate the intersection of these two disciplines and examine the way in which demographic trends associated with decreasing levels of inbreeding may influence genomic structure and how this may affect medical genetics research. By examining two age-heterogeneous populations of outbred North Americans, we were able to ascertain genetic changes occurring over the past century that have been likely brought about by recent increases in mobility, urbanization, and population admixture. Using multiple measures of the genomic manifestations of distant consanguinity, we showed significant trends towards decreasing levels of autozygosity and more marginal inbreeding coefficients as study participant birth years neared the chronological present day. We believe this finding is particularly important, as decreasing autozygosity and less homozygosity genome-wide may help to slightly reduce the burden of rare recessive diseases in the future.

## Introduction

Rates of travel and migration within North America have increased substantially over the past century due to advancements in infrastructure and technology. It has been hypothesized that this ease of travel and globalization has shaped the demographic structure of both North American and world populations in recent generations. Within the past two centuries, population growth, admixture and expansion have been rapid, causing increasing genetic variation in many populations [1],[2].

In our research, we have investigated how demographic trends in the past century have been recapitulated in quantifiable genetic changes, and how this may impact medical genetics and genetic diseases. We focused our study on genome-wide rates of autozygosity and measured tracts of extended homozygosity in two age-heterogeneous samples of North Americans to estimate the genetic effect possibly attributable to demographic change.

We measured autozygosity in the form of runs of extended homozygosity (ROHs). We analyzed these extended tracts of homozygosity genome-wide, showing a strong positive association between increasing chronological age and increasing rates of autozygosity. These homozygous runs were used to quantify consanguinity in our analysis populations. We also utilized a modified inbreeding coefficient to quantify decline in the proportion of excess homozygosity outside of linkage disequilibrium. This modified inbreeding coefficient, has been calculated using data that has had all neighboring single nucleotide polymorphisms (SNPs) that are in linkage disequilibrium (LD) with each other removed (LD pruned data), and is referred to as F_ld_. Our results show that older members of the population have a tendency to possess more homozygous runs, which comprise a higher percentage of the total genomic length, than those found in younger participants. These older participants also exhibit a larger proportion of excess homozygosity based on estimates from inbreeding coefficients.

The homozygous runs representing autozygosity in relatively outbred populations may also be highly relevant in disease gene discovery. The mapping of these regions on a genome-wide scale could help to identify low-frequency variants associated with complex disease [3],[4]; therefore helping to alleviate the methodological constraints of the common-disease/common-variant mode of inheritance that is generally utilized in whole-genome associations studies [5]. Genetic effects of consanguinity have been shown to be associated with epistatic effects at disease susceptibility loci causing reduced resistance to environmental risk factors and infectious diseases [6],[7]. Epidemiological studies, and animal models, have provided empirical evidence that consanguinity is a risk for complex diseases such as high blood pressure, cancers, osteoporosis, schizophrenia, epilepsy and depression [1], [6], [8]–[10].

## Results

We first measured the runs of homozygosity in our discovery population comprised of a cohort of controls compiled by the Coriell Institute, to be a representative sample of neurologically normal North Americans of European descent. We generated summary descriptive statistics for all 809 individuals aged 19–99 that quantified mean and standard deviations for number of ROHs, total percentage of the genome in ROHs (%ROH) and average ROH length (Table 1). We then compared these measures across strata of ∼20 year age groups. In comparisons of the youngest and oldest age strata, significant differences exist in all measures of homozygosity. The oldest age group (estimated current age ≥80 years) presented larger, more frequent ROHs, causing more of the genome to be comprised of ROHs than the youngest age group (estimated current age ≤39 years). These differences were significant (|t|>2.5, p-value≤0.01) for all ROH measures, although the greatest difference appeared in comparing %ROH between the two age groups (|t| = 3.53, p-value = 0.0005). Differences in F_ld_ were suggestive (|t| = 1.91, p-value = 0.056). This illustrates possible generational differences in autozygosity in an outbred population of unrelated individuals (Table 2).

**Table 1:** Descriptive statistics for total population.

Cohort	Coriell	BLSA
N	809	477
Percent female	58.0	47.3
Current age (years)	61.7 (16.7)	68.3 (13.7)
Number of ROHs	28.6 (5.9)	27.6 (5.4)
% Genome in ROHs	1.6 (0.6)	1.5 (0.5)
Average ROH length (Mb)	1.5 (0.3)	1.4 (0.2)
F_ld_ (%)	0.7 (1.9)	−0.3 (1.2)

All measures in mean (standard deviation).

**Table 2:** Measures of autozygosity vary by 20-year age groups.

Age range (years)	19–39		40–59		60–79		80–99	
Cohort	Coriell	BLSA	Coriell	BLSA	Coriell	BLSA	Coriell	BLSA
N	104	16	198	109	395	239	112	113
Current age (years)	31.9 (4.9)	33.5 (3.6)	49.7 (5.8)	53.7 (4.8)	68.9 (5.7)	69.9 (6.1)	85.0 (4.4)	84.9 (3.8)
Number of ROHs	27.2 (5.4)*	26.3 (4.3)	28.4 (5.9)	26.4 (4.9)$	28.9 (5.9)	27.8 (5.9)	29.5 (5.8)*	28.4 (5.6)$
% Genome in ROHs (%ROH)	1.44 (0.3)*	1.36 (0.2)	1.57 (0.5)	1.41 (0.3)$	1.61 (0.7)	1.51 (0.5)	1.67 (0.6)*	1.58 (0.5)$
Average ROH length (Mb)	1.40 (0.1)*	1.36 (0.1)	1.46 (0.3)	1.41 (0.1)$	1.47 (0.4)	1.43 (0.2)	1.48 (0.3)*	1.47 (0.3)$
F_ld_ (%)	0.39 (2.4)*	−0.29 (0.9)	0.57 (1.8)	−0.60 (1.3)^x^	0.69 (1.7)	−0.21 (1.1)	0.99 (2.2)*	−0.07(1.2)^x^

All measures in mean (standard deviation) format when applicable.

*: indicates significant difference of within row-comparison of measures in Coriell samples (p-value<0.05).

$ indicates significant difference within row-comparison of measures in BLSA samples (p-value<0.05).

x indicates suggestive (p-value<0.07).

The trend of increasing autozygosity associated with chronological age remained significant in a number of linear regression models, each model incorporating different covariates (Table 3). In multivariate regression models following up the initial results, the associations of chronological age and both the number of ROHs and the %ROH were unattenuated by the introduction of statistical adjustments for either observed or expected homozygosity outside of linkage disequilibrium, F_ld_ (standardized β≥0.10, t-statistic≥2.5, p-values≤0.01). Trends showing a positive association between F_ld_ and chronological age were also significant (standardized β≥0.08, t-statistics≥2.14, p-value≤0.033). This association between F_ld_ and chronological age was relatively unattenuated in models adjusted for average ROH length. The weakest associations with chronological age occurred when examining the linear association with average ROH length. The effect size of this association with average ROH length was small, with standardized beta-coefficients varying between 0.06 and 0.07 (t-statistics between 1.77 and 2.12), although still below our *a priori* significance threshold of p<0.05 for two of the models. All models for the association between chronological age and average ROH length were at least borderline significant (p-value<0.10).

**Table 3:** Linear regression models showing autozygosity measures are positively correlated with chronological age (standardized to 2008), reporting standardized β coefficients and p-values.

	Number of ROHs				Percent Genome ROH (%ROH)				Average ROH Length				Inbreeding Coefficient (F_ld_)			
Study	Coriell		BLSA		Coriel		BLSA		Coriell		BLSA		Coriell		BLSA	
Model 1	0.12,	0.001	0.16,	<0.001	0.12,	0.001	0.18,	<0.001	0.07,	0.035	0.11,	0.016	0.08,	0.015	0.15,	0.001
Model 2	0.10,	0.005	0.14,	0.003	0.10,	0.005	0.15,	0.002	0.06,	0.066	0.09,	0.047	*NA*		*NA*	
Model 3	0.12,	0.001	0.16,	<0.001	0.11,	0.001	0.18,	<0.001	0.07,	0.038	0.11,	0.016	*NA*		*NA*	
Model 4	0.10,	0.006	0.13,	0.004	0.10,	0.006	0.14,	0.003	0.06,	0.077	0.09,	0.058	0.08,	0.033	0.14,	0.021
R^2^, maximum	0.12		0.08		0.10		0.10		0.03		0.03		0.01		0.02	

Model 1 is unadjusted.

Model 2 includes covariate of observed homozygosity outside of LD.

Model 3 includes covariate of expected homozygosity outside of LD.

Model 4 includes covariate of F_ld_ for dependent variables of number of ROHs, %ROH and average ROH length, for the models using F_ld_ as the dependent variable average ROH length was used as a covariate.

**Figure 1:**
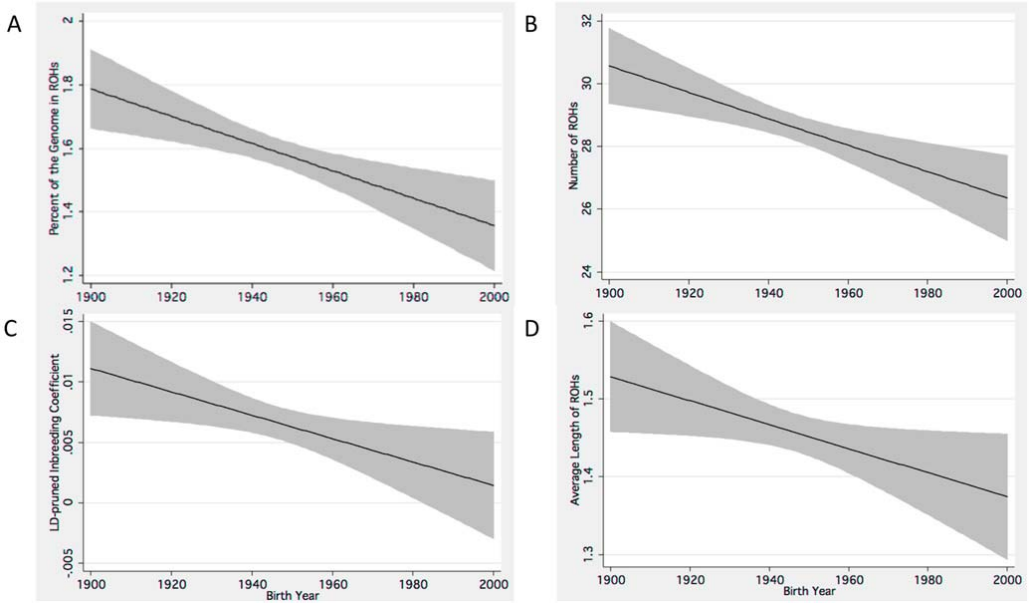
Linear predictive models show declining measures of autozygosity and F_ld_. Estimates of trends for declining autozygosity in individuals born in the twentieth century. These predictive models include decreasing (A) %ROH, (B) number of ROHs, (C) F_ld_ and the (D) average length of ROHs. The black lines represents linear predictive trends, and the grey shaded area represents the 95% confidence interval for these estimates.

The predictive models based on the results of our regression analyses forecasts a multi-faceted trend for increasing autozygosity as years since birth increases (from the temporal present). These models, summarized in Figure 1, describe patterns of autozygosity and excess homozygosity having decreased over the twentieth century. Based on these hypothetical models that include data imputed to additional years outside of the 80 birth year range for participants in this study, the number of ROHs has decreased by 14.0% over the past 100 years; while the %ROH and average run lengths have also decreased by similar factors, 24.3% for the former and 10.5% for the latter. F_ld_ has decreased by a factor of 30.5% in our models.

### Replication

A subset of participants from the Baltimore Longitudinal Study of Aging (BLSA), were selected to replicate these associations from our analyses of the Coriell Control cohort. These participants were selected based on their similarity to the Coriell cohort used in the initial analyses. The results from the BLSA cohort supported all of our hypotheses tested in the initial discovery cohort.

In the BLSA cohort, significant differences in rates of homozygosity exist when comparing participants between the standardized chronological ages of 40–59 years to those aged 80–99 years. These two, more demographically similar, age groups were compared to stringently test replication due to the relatively few BLSA samples in the 19–39 year old cohort (N = 19) that would have limited statistical power for comparisons. Results were consistent to those in the initial analyses of the Coriell Control cohort, with t-tests showing significant differences between age strata for number of ROHs (|t| = 2.8, p-value = 0.0051), %ROH (|t| = 3.1, p-value = 0.0022), and average ROH length (|t| = 2.0, p-value = 0.0485). The younger participants were generally less homozygous than the older strata, with significant differences in F_ld_ (|t| = 3.15, p-value = 0.0018).

Linear regression models of age-associated decrease in measures of autozygosity were also utilized to test the validity of our initial results. Identical regression models as those used in the Coriell cohort were used in the BLSA cohort. The smaller BLSA cohort successfully replicated all trends found to be significant in the analyses of the Coriell cohort. These models were generally more significant in the replication analyses than in the initial discovery cohort (Table 3).

## Discussion

This research has definitively shown the existence of a trend for decreasing autozygosity with younger chronological age in the North American population of European ancestry. The ROHs we identified, larger than 1 Mb, are clearly representative of autozygosity due to distant consanguinity in our outbred populations, and not chromosomal abnormalities or common copy number variants [3],[11],[12]. Using our predictive models of decreasing F_ld_, we show a quantifiable decrease in consanguinity over the twentieth century. Based on data provided in Carothers et al [13], this decrease in F_ld_ found in our discovery population is on the order of individuals transitioning from a single inbreeding loop 4–5 generations prior, to no inbreeding loops within <6 generations. We postulate that the increased mobility, urbanization and outbreeding in North America in the last century has led to less consanguinity (and thus less homozygosity and homogeneity) in younger individuals [1],[2].

We have shown a weaker association with chronological age for the measured average ROH length. It is possible that since populations that are becoming more outbred, less consanguinous and more heterogeneous recombination could fracture ROHs into smaller segments, for which a robust measure such as %ROH would be less affected than average ROH size. This could contribute to higher variation in average run length measurements, resulting in increased variation in the measure causing more possible type I error and decreased statistical power when compared to %ROH.

With extended regions of homozygosity decreasing in size and becoming less frequent, this structural genomic trend may have some latent effect on public health, as well as the recently developed methods for genome-wide association studies (GWAS). Theoretically, an excess of ROHs and excess homozygosity (identified using the linkage pruned inbreeding coefficient, F_ld_) may increase the chances of rare recessive genetic diseases. The trends shown in this research may have a larger impact in modifying the epigenetic, epistatic and polygenic pathways that influence many complex traits [6],[7]. This is particularly of interest when considering the rates at which partially recessive alleles may decline in frequency and reduce phenotypic variation in complex polygenic traits.

Our results show that if demographic trends continue towards a globalized, urbanized and more freely mobile world, populations will become less consanguineous.

## Materials and Methods

### Genotyping and Quality Control

This initial genome-wide analysis was undertaken on a subset of the 828 unrelated clinical controls from the National Institute of Neurological Disorders and Stroke (NINDS) funded Neurogenetics repository at the Coriell Institute. These samples were collected by Coriell to be used as convenience genetic controls, representative of the North American population of varied European descent. The replication cohort for this study was taken from the Baltimore Longitudinal Study of Aging, a community based longitudinal study of aging currently in its 50^th^ year of follow up.

Individuals from both cohorts were genotyped concurrently at the National Institute on Aging's Laboratory of Neurogenetics (LNG) using the Illumina Infinium technology (Illumina Inc., San Diego, CA). The assays used for genotyping included were the Infinium II HumanHap550 v. 1, Infinium II HumanHap550 v. 3, or a composite of Infinium HumanHap300 and Infinium II 240S. By combining the genotype data from Infinium HumanHap300 and Infinium II 240S assayed participants, to an equivalent level of genomic coverage as the Infinium II HumanHap550 assays, we were able to standardize participant data across a total of 545,066 single nucleotide polymorphisms genotyped on the Illumina platforms. The raw genotype data were stored and quality controlled using GERON genotyping (http://neurogenetics.nia.nih.gov), an intranet repository for genotype data created on the Illumina platform.

All samples from both cohorts were first quality controlled for a minimum of a 97% successful genotype call rate. Any samples failing this initial quality control step were re-genotyped using a new DNA aliquot until a 95% successful call rate was achieved. 13 of the initial DNA samples from our neurological control population were ultimately excluded due to consistent call rates below our inclusion threshold of 97%. Of the 848 participants from BLSA that were genotyped who based on available data were not self-reported African American, 34 samples had call rates below 97%. SNPs with minor allele frequencies less than 5% and departures from Hardy-Weinberg equilibrium (HWE test, p<0.01) and missingness per SNP greater than 5%, were excluded from further analyses. PLINKv1.0.1 was used to carry out sex-checks based on heterozygosity of the X chromosome genotypes were used to exclude 4 participants from the Coriell cohort whose self-reported sex did not match that presented in the genotypic data ([14], http://pngu.mgh.harvard.edu/~Purcell/plink/). 14 of the samples from the BLSA cohort were excluded due to X chromosome heterozygosity inconsistent with self-reported sex. Blinded sex-checks were carried out for all samples using the default function for genotypic estimates of gender in the Illumina Beadstudio package (Illumina Inc., San Diego, Caliornia). The only conflicting genders reported by the Beadstudio results were identical to those reported by PLINK.

All samples utilized from both studies underwent further quality control procedures to check for any indications of population stratification or substructure. Identical by descent (IBD) analyses were used to identify repeated samples or distantly related individuals, with an apriori exclusionary criteria of sharing at most a 2.0% proportion of the genotyped SNPs as identical by descent in any pair-wise combinations of samples. This strict exclusionary criteria is necessary, as including any participants that are related within recent generations could bias our analyses and lead to a non-independence of measures of autozygosity. Probands from groups of related individuals were randomly selected for inclusion in the analyses. This IBD filtering called for the removal of one duplicate sample from the Coriell population. This filter eliminated 101 cryptically related individuals from the BLSA. The high number of related individuals in the BLSA is to be expected as a result of recruitment patterns in the study. The BLSA itself is a cohort of volunteers initially recruited from a group of retired Federal scientists, who subsequently recruited friends and family members. Family members of early participants in the BLSA were given priority enrollment during the course of the study.

Identical by state distances were generated using multi-dimensional scaling for the remaining samples in an attempt to identify population outliers not already considered during the admission/adjudication of the participants. The distribution was standardized on HapMap samples used to aid in the detection of stratification and outliers. One participant was removed from the Coriell cohort of neurological controls due to ancestry consistent with African samples. There were no identifiable sub-population clusters or outliers apparent within the European American samples in the multidimensional scaling analyses of the Coriell dataset. When the Coriell cohort, BLSA cohort, and HapMap samples underwent combined multidimensional scaling analyses (using 410,834 shared quality controlled SNPs as a basis for comparison), additional outliers for possible stratification effects were removed from the BLSA cohort, with 222 samples being more than two standard deviations from the combined population mean for any of the four components of the MDS model. BLSA is a relatively ethnically diverse population, compared to the Coriell samples. Beginning in 1990, a conscious effort was made to recruit African Americans, while Asians, Latin Americans and participants from other ethnic groups were also recruited in recent decades. This deviation from mean component measures in BLSA during the quality control process may be due to some slight level of additional cryptic relatedness or population admixture/stratification not seen in the discovery cohort samples. All genotyping quality assessments, sex-checking, IBS and IBD calculations were carried out using PLINK v1.0.1. A summary of quality control results can be found in Table S1 in the supplemental materials.

### Coriell Popualtion

After the quality control process, we were left with an analytic population of 809 neurologically normal participants genotyped at 476,962 SNPs. These participants were sampled between the ages of 15 and 95 years old (age range = 80 years, mean age at sampling = 61.7 years old, standard deviation = 16.7 years). However, records show a 6 year period over which these controls were adjudicated and sampled. There was no statistical correlation between the dates of sample collection and participant age (Pearson correlation, p-value>0.05), this suggests no sampling bias with regard to age at collection. This data allowed for the calculation of the participants' estimated current chronological age (within less than1 year) standardized to the year 2008. Chronological age refers to the participant's calculated current age, regardless of death, and ranges between 19–99 years of age (mean age of 61.7 years±16.8). The population is comprised of 57.9% female participants.

### BLSA Population

477 participants from the BLSA were selected for replication purposes after passing similar quality control to the Coriell cohort, including the removal of genotyping failures, population outliers and cryptically related samples. These samples were individually standardized to 450,364 quality controlled SNPs. BLSA samples in the replication population possessed a population standardized mean chronological age of 68.3 (S.D. = 13.7). With regard to autozygosity measures, the BLSA samples were slightly lower than those of the Coriell cohort in both the entire population and the four age strata (Tables 1 and 2). The BLSA samples also exhibited ∼1% more excess heterozygosity than the Coriell cohort, based on mean F_ld_ calculations. This may be due to the fact that the BLSA cohort is derived from an urban dwelling population based in Baltimore, MD. The BLSA cohort was comprised of slightly more males than the Coriell cohort, but this should not be a factor in the replication as all analyses were confined only to the 22 autosomes.

### Quantifying Autozygosity using Runs of Homozygosity

We utilized the PLINK v1.0.1 toolkit to identify runs of homozygosity. Primary criteria for inclusion of a genomic region into a homozygous run are the region must be at least 1 megabase in length and contain at least 50 adjacent SNPs (per Mb) with homozygous genotype calls. This robust size and SNP density threshold for inclusion into ROHs allows for the algorithmic exclusion of copy number variants, centromeric and SNP-poor regions. The density requirement of at least 50 SNPs per Mb is based on an apriori genome-wide coverage target of ∼500,000 quality controlled SNPs in analytic populations. This requirement of at least 50 SNPs per Mb is similar to the requirements for ROHs found in the Gibson et al., 2005 analysis of runs of homozygosity in HapMap Phase II data [15]. A sliding window of 50 SNPs was used to identify these runs, and included no more than 2 SNPs with missing genotypes and 1 possible heterozygous genotype. These analyses were limited to the 22 autosomal chromosomes. Identical parameters were used to generate these measures in the BLSA cohort as were used in the Coriell cohort.

Our metrics for comparing rates of autozygosity among the participants in this study were able to be calculated after identifying the ROHs. Our primary measures of autozygosity include: total percentage of the genome included in ROHs and the average length of ROHs. The total percentage of the genome included in ROHs was calculated by summing the length of each individual ROH per participant. This summed length of identified ROHs was then divided by a factor of 2,645 and subsequently converted to a percent by multiplying the dividend by 100. The division by 2,645 in the generation of the %ROH measure is based on the number of megabases covered by SNPs included in the Infinium HumanHap 550v.1 and 550v.3 assays used to generate our genome-wide datasets. This estimate of coverage of 2,645 Mb was calculated by summing the distance between the first and last available SNP of each chromosomal arm for each of the 22 autosomes. The average length of ROHs was calculated by dividing the total length by the number of ROH segments per participant. Both of these measures are expressed in Mb.

### Linkage-Pruned Inbreeding Coefficient (F_ld_) and Covariates

As ROHs are associated with regions of high linkage disequilibium, we also attempted to examine rates of homozygosity outside of LD. To calculate the additional measure of F_ld_, we created LD pruned versions of each of the genome-wide datasets. We accomplished this by algorithmically excluding SNPs in LD with neighboring SNPs to create the LD pruned datasets, and then calculating inbreeding coefficients based on the remaining data.

Using data from all 809 participants in our analysis population (and a separate-identical analysis for all 477 participants in our replication study), we calculated variance inflation factors (VIF) for each of the possible pairwise combinations of SNPs within a sliding window of 50 SNPs (with 5 SNP overlaps per window). We excluded all SNPs with a VIF>1.05 within each sliding window. This VIF threshold corresponds to a maximum multiple correlation coefficient representative of ∼1% co-linearity of genotype calls with any other SNP in the sliding window of analysis. This stringent variance threshold allowed us to trim the genome-wide dataset for the Coriell cohort to 48,902 SNPs dispersed relatively evenly across the 22 autosomes, and to 34,307 autosomal SNPs in the BLSA cohort.

We calculated expected rates of homozygous calls per participant based on HWE expectations of genotype frequencies using the LD-pruned datasets. We then calculated cohort specific observed rates of homozygosity within the LD-pruned datasets, expressed as a summed count of homozygous genotypic calls per participant. Using PLINK, we then calculated single population inbreeding coefficients (F statistics) to summarize the proportion of homozygous genotypes differing from our HWE based expectations per participant. These F statistics, based on calculations carried out on the datasets containing only SNPs not in LD with each other, comprise the summary measure we refer to as the LD pruned inbreeding coefficient (F_ld_). We converted this to a percentage in our tables for ease of comparison, although actual F_ld_ coefficients are used in the predictive models. In our randomly selected populations of unrelated individuals, the F_ld_ values we calculated are a proxy for the occurrence of excess homozygosity on a genome-wide level. The F_ld_ statistic allows for an accurate assessment of autozygosity outside of linkage disequilibrium, without being adversely affected by low SNP density after removing SNPs in regions of LD. The linkage pruned sub-sets of the genotypic data was used for the calculation of observed and expected rates of homozygosity outside of LD as well of the generation of the inbreeding coefficients that comprise the F_ld_ statistic. A discussion of the use of the LD-pruned data to construct ROHs may be found in the supplemental materials in Text S1.

### Statistical Analyses

Descriptive statistics were generated for all variables involved in analyses. These include counts, means and standard deviations for all three measures of autozygosity (number, %ROH and average run length) and F_ld_ to be used as dependent variables, as well as for the primary predictor variable of estimated chronological age. These measures were all relatively normally distributed in our population of neurological controls.

Generational differences were estimated by sorting participants into 4 age strata based on 20 year intervals. Descriptive statistics were calculated again to compare mean variation in measures of autozygosity and F_ld_. Differences in mean measures of autozygosity and F_ld_ between the oldest (estimated current age of 80–99 years) and youngest (estimated current age of 19–39 years in the Coriell cohort) generations were compared using a basic two-way t-test for each of the autozygosity measures.

Linear regression models were constructed in order to investigate possible associations between chronological age and autozygosity measures. Similar models were used to evaluate the association between F_ld_ and chronological age. Separate regression models were constructed for dependent variables of number of ROHs, %ROH, average ROH length and F_ld_. These models were initially adjusted for gender only (gender was not a statistically significant term in any models, p-value>0.05), although to create more parsimonious models, gender was not included. Additional covariates of observed and expected rates of homozygosity outside of LD were added to the second and third sets of models respectively to further scrutinize and follow-up the initial results. F_ld_ was used as a covariate in the fourth model set to account for the possible confounding effect of chance homozygosity outside of LD in the examinations of trends involving measures of autozygosity derived from ROHs. Subsequent regression models evaluating the trend for increasing F_ld_ with chronological age were adjusted for average ROH length. Additional regression models investigating associations in combined cohorts are described in the supplemental materials in Text S1 and Figure S1.

A second set of linear regression models were created to investigate the possible multiplicative effect of age, by using age^2^ as the primary predictor of increasing autozygosity measures (gender adjusted) or F_ld_. However, none of these models that incorporated age^2^ showed a stronger association with the measures of autozygosity, as the standardized-beta-coefficients and r^2^ values were actually smaller than those in the previous models of linear age. These models are not included in the manuscript as they add no additional pertinent information, but are available upon request.

Linear predictive models of autozygosity decrease over the twentieth century were extrapolated from regression models based on the Coriell discovery cohort. These models estimate decreasing rates of autozygosity and excess homozygosity as time progresses. These are based on the regression coefficients from the original un-adjusted models of chronological age predicting demographic change in the total number of ROHs, %ROH, average ROH lengthand F_ld_. These models provide estimates of time dependent means and confidence intervals for both measures. Percent change over 100 years was estimated for each measure using these models. All estimates of percent change were based on a minimum value of zero except for F_ld_, when a scalar minimum for the calculation based on the lowest value within the 95% confidence interval of the predictive model (F_ld_ = −0.0031) was used.

## Supporting Information

Figure S1 Regression plots showing trends for %ROH and F_ld_ decline as participant birth year increases. Regression plots showing trends in declining autozygosity in combined cohorts. Panel A shows decreasing percent of the genome contained in ROH as birth year increases. Panel B shows decreasing F_ld_ as birth year increases. Red lines represent linear trends adjusted for study site. Points represented by the symbol ‘+’ indicate Coriell samples, points represented by the symbol ‘x’ indicate BLSA samples.
(1.56 MB TIF)

Table S1 Summary of exclusions made in data cleaning process.
(0.03 MB DOC)

Text S1 Supplemental materials.
(0.04 MB DOC)
