# Supplementary figures and images for: Measures of Autozygosity in Decline: Globalization, Urbanization, and Its Implications for Medical Genetics
Source: PLoS Genet. 2009 Mar 13;5(3):e1000415. doi: 10.1371/journal.pgen.1000415 (PMC2652078; doi:10.1371/journal.pgen.1000415)

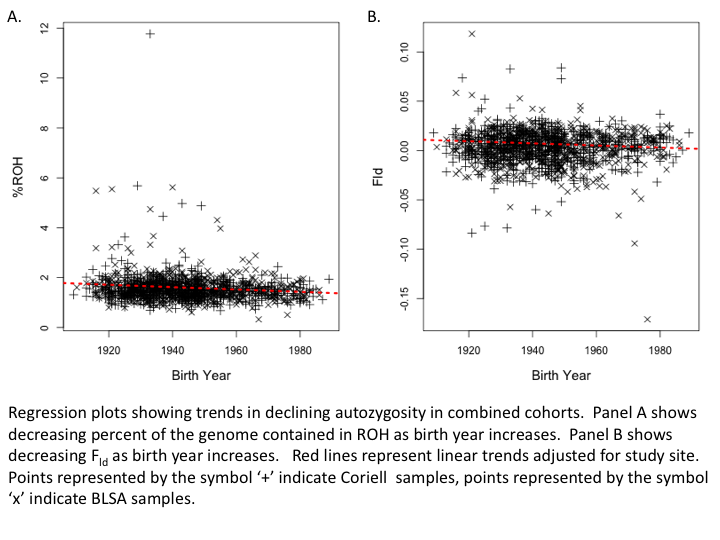

Supplement: Figure S1 — Regression plots showing trends for %ROH and Fld decline as participant birth year increases. Regression plots showing trends in declining autozygosity in combined cohorts. Panel A shows decreasing percent of the genome contained in ROH as birth year increases. Panel B shows decreasing Fld as birth year increases. Red lines represent linear trends adjusted for study site. Points represented by the symbol ‘+’ indicate Coriell samples, points represented by the symbol ‘x’ indicate BLSA samples. (1.56 MB TIF) [file pgen.1000415.s001.tif]
