# Supplementary material for: Measures of Autozygosity in Decline: Globalization, Urbanization, and Its Implications for Medical Genetics
Source: PLoS Genet. 2009 Mar 13;5(3):e1000415. doi: 10.1371/journal.pgen.1000415 (PMC2652078; doi:10.1371/journal.pgen.1000415)
Supplement: Table S1 — Summary of exclusions made in data cleaning process. (0.03 MB DOC) [file pgen.1000415.s002.doc]

| **Study** | **Coriell** | **BLSA** |
| --- | --- | --- |
| **Sample Exclusions** |  |  |
| Minimum call rate of 97% | 13 | 34 |
| Sex discordance | 1 | 14 |
| Relatedness/sample duplication | 1 | 101 |
| Population outlier | 1 | 222 |
| **SNP Exclusions** |  |  |
| Concurrent exclusion based on |  |  |
| HWE p<0.01, MAF<5% | 68104 | 94702 |
| and missingness per SNP > 5% |  |  |
| **Final sample sizes after exclusions** |  |  |
| N | 809 | 477 |
| SNPs | 476962 | 450364 |
