# Supplementary material for: Measures of Autozygosity in Decline: Globalization, Urbanization, and Its Implications for Medical Genetics
Source: PLoS Genet. 2009 Mar 13;5(3):e1000415. doi: 10.1371/journal.pgen.1000415 (PMC2652078; doi:10.1371/journal.pgen.1000415)
Supplement: Text S1 — Supplemental materials. (0.04 MB DOC) [file pgen.1000415.s003.doc]

**SUPPLEMENTAL MATERIALS**

*ROHs Outside of Linkage Disequilibrium*

To generate hypotheses regarding the effect of linkage disequilibrium on ROHs, we identified ROHs in the LD-pruned Coriell dataset. We used nearly identical criteria for ROH identification in this analysis as in the primary genome-wide analyses. The only change made to the ROH identification requirements is a proportionate decrease in necessary SNP density for a region to achieve membership in a ROH. This is due to ~90% of the previous density of SNP coverage being excluded by the LD pruning algorithm. For membership in an LD-pruned ROH, a minimum density of only 5 SNPs per Mb was necessary.

ROHs were identified, with a population average of 22.1 ROHs per participant (standard deviation [SD] = 4.9), an average of 1.0% of the genome composed of ROHs (SD = 0.3), and an average ROH length 1.2 Mb (SD = 0.1). These LD-pruned ROHs were then used a dependent variables in a series of univariate regression analyses to assess any possible associations between LD-pruned ROHs and standardized participant age. The regression model showed a significant positive association between %ROH and increasing standardized age remained significant (|t| = 2.25, p-value = 0.025) , as well as for average ROH length being associated with increasing standardized participant age (|t| = 2.81, p-value = 0.005). The only association to be attenuated by LD-pruning datasets prior to calculating ROHs is number of ROHs, which showed and insignificant association with standardized age (|t| = 1.25, p-value = 0.210).

The methods used for linkage pruning may have had an affect on our results. While LD-pruning will decrease the false positive rate for successful identification of ROHs, it may also cause an increase in the false negative rate. Particularly if a dataset is excessively LD-pruned, low SNP density may contribute to excessive rates of false negatives in the identification of ROHs [15].

*Regression of Combined Cohorts*

Data for participant birth year, %ROH and F_ld_ from both cohorts were combined for a series of follow up analyses for our primary measures of interest (%ROH and F_ld_). The mean %ROH for the combined samples was 1.56, with a standard deviation of 0.56. The mean combined F_ld_ for both cohorts was 0.003, with a standard deviation of 0.017. Multivariate regression models were generated to estimate trends for decreasing autozygosity in a pooled group of samples. Membership in the BLSA cohort was the only covariate in these two models and was coded as a binomial indicator. These analyses used birth year as a predictor of decreasing %ROH, showing a significant association (|t| = 4.83, p-value = <0.001). F_ld_ also showed a significant decrease correspoding to increasing birth year (|t| = 3.60, p-value < 0.001). Results for these regression models are show in Figure S1.

A second set of models excluding individuals with %ROH or Fld estimates outside of 2 standard deviations for either of the traits were undertaken as follow-up to the initial combined models. Membership in BLSA was the only covariate in these follow-up models. Both associations remained significant after exclusions, showing normal decline associated with increasing birth year in %ROH and F_ld_ ( |t| = 4.53, p-value < 0.001 for regression including %ROH, |t| = 2.81, p-value = 0.005 for the model including F_ld_).
